# Supplementary material for: SyntheMol-RL: a flexible reinforcement learning framework for designing easily synthesizable antibiotics
Source: Mol Syst Biol. 2026 Apr 23;22(6):833–67. doi: 10.1038/s44320-026-00206-9 (PMC13230741; doi:10.1038/s44320-026-00206-9)
Supplement: Supplementary file 1 — Appendix [file 44320_2026_206_MOESM1_ESM.pdf]

Appendix for “SyntheMol-RL: a flexible reinforcement learning framework for designing easily synthesizable antibiotics”

Table of Contents

1 Appendix Extended Discussion 2
2 Appendix Table S1 5
3 Appendix Table S2 5

Extended Discussion

We compared SyntheMol-RL to two state-of-the-art generative models for small molecule drug design: GFlowNet (Bengio *et al*, 2021) and REINVENT 4 (Loeffler *et al*, 2024). GFlowNet employs an RL-like generative model with sampling for diverse molecule generation, which inspired some of the elements of SyntheMol-RL, and can be trained with multiple objectives (Jain *et al*, 2023). REINVENT 4 is an RL model that designs molecules by generating SMILES and can also be trained in multi-objective form. We trained both GFlowNet and REINVENT 4 to design molecules using four reward functions: (1) our antibacterial activity Chemprop-RDKit model, (2) our aqueous solubility Chemprop-RDKit model, (3) the synthetic accessibility score SAScore (Ertl & Schuffenhauer, 2009), and (4) molecular weight. The former two rewards are the same models used to reward SyntheMol-RL, while the latter two rewards account for the fact that GFlowNet and REINVENT 4 models do not inherently generate easily synthesizable molecules. The models were run long enough to generate approximately 10,000 molecules to match SyntheMol-RL. The GFlowNet model ran for 4 hours and 45 minutes on 8 CPUs and 1 GPU and used 4.20 GB of memory. The REINVENT 4 model ran for 26 minutes on 8 CPUs and 1 GPU and used 3.33 GB of memory.

The molecules generated by GFlowNet and REINVENT 4 were filtered to obtain hits using the same filters as for the SyntheMol-RL molecules (predicted antibacterial activity ≥ 0.5, predicted log solubility ≥ -4, novelty, and diversity) along with two filters for synthesizability: SAScore ≤ 4 and molecular weight ≤ 600. These latter two filters are not needed for SyntheMol-RL, which inherently generates small, easily synthesizable compounds. Based on these filters, RL-Chemprop generated 186 hits (total = 10,983 molecules), RL-MLP generated 205 hits (total = 9,228 molecules), GFlowNet generated 1,152 hits (total = 10,304 molecules), and REINVENT 4 generated 36 hits (total = 9,840 molecules after filtering out 160 invalid SMILES from the

original 10,000 molecules). Fig. EV7A-B shows the distribution of antibacterial prediction scores and predicted log solubilities among the hits.

Although the GFlowNet model generated the most hits (1,152), the GFlowNet molecules contain complex multi-ring structures that would likely suffer from poor whole cell activity (Richter *et al*, 2017) and synthesizability (Ertl & Schuffenhauer, 2009) compared to the SyntheMol-RL compounds, despite the promising predicted SAScores (Dataset EV12, Fig. EV7C-E). This result is likely because the Chemprop antibacterial model assigns higher scores to molecules with more antibacterial functional groups that each individually score highly, so GFlowNet learns to add as many of those groups (in this case rings) as possible, even if this produces ineffective and difficult-to-synthesize compounds. In contrast, REINVENT 4 generated the fewest hits, but these hits do not possess the same multi-ring structures preferred by GFlowNet (Fig. EV7F). However, the REINVENT 4 compounds are also not clearly easy to synthesize, despite the high SAScores. Both GFlowNet and REINVENT 4 generated relatively diverse and novel molecules (Fig. EV7G-I).

To confirm the real world synthesizability of the GFlowNet and REINVENT 4 compounds, we selected the top 20 hits from each model by antibacterial prediction score and consulted medicinal chemists at Enamine and WuXi. For the GFlowNet compounds, Enamine was only able to offer synthesis of 4 of the 20 compounds, and they would cost 26-62x more and take 2-6x longer to synthesize than the SyntheMol-RL compounds from the Enamine REAL Space, which cost around $110-170 per compound and took 3-5 weeks to synthesize. WuXi was unable to offer a fixed quote for any of these 20 compounds since the "chemistry is [a] challenge". They instead offered to have four chemists work for three months to attempt synthesis, which would

cost 17x more and take 2x longer to synthesize than the SyntheMol-RL compounds from the WuXi GalaXi, which cost $200 per compound and took 6-8 weeks to synthesize. For the REINVENT 4 compounds, Enamine was only able to offer synthesis for 10 of the 20 compounds, and they would cost 2-37x more and take up to 4x longer to synthesize than the SyntheMol-RL compounds from the Enamine REAL Space. WuXi was able to offer synthesis for 15 of the 20 compounds, and they would cost 2-17x more while taking a similar amount of time to synthesize as the SyntheMol-RL compounds from the WuXi GalaXi.

Collectively, these results highlight the strong value of SyntheMol-RL. While other molecular design models may generate compounds with good predicted properties *in silico*, their reliance on imprecise synthesizability predictors results in molecules that are difficult and expensive to synthesize, thus hindering their real-world value for drug discovery applications. SyntheMol-RL, in contrast, can translate promising *in silico* compound designs to actual, synthesized molecules for rapid and inexpensive laboratory validation.

Extended Discussion References

Bengio E, Jain M, Korablyov M, Precup D & Bengio Y (2021) Flow Network based Generative Models for Non-Iterative Diverse Candidate Generation. In *Advances in Neural Information Processing Systems* pp 27381–27394. Curran Associates, Inc.

Ertl P & Schuffenhauer A (2009) Estimation of synthetic accessibility score of drug-like molecules based on molecular complexity and fragment contributions. *J Cheminformatics* 1: 8

Jain M, Raparthy SC, Hernández-García A, Rector-Brooks J, Bengio Y, Miret S & Bengio E (2023) Multi-Objective GFlowNets. In *Proceedings of the 40th International Conference on Machine Learning*, Krause A Brunskill E Cho K Engelhardt B Sabato S & Scarlett J (eds) pp 14631–14653. PMLR

Loeffler HH, He J, Tibo A, Janet JP, Voronov A, Mervin LH & Engkvist O (2024) Reinvent 4: Modern AI–driven generative molecule design. *J Cheminformatics* 16: 20

Richter MF, Drown BS, Riley AP, Garcia A, Shirai T, Svec RL & Hergenrother PJ (2017) Predictive compound accumulation rules yield a broad-spectrum antibiotic. *Nature* 545: 299–304

Appendix Table S1. Pairwise p-values for two-tailed, two proportion z-tests comparing number of hits discovered between models.

	RL-Chemprop	RL-MLP	MCTS	VS-Chemprop
RL-Chemprop		1.49 x 10^-54	4.54 10^-138	0
RL-MLP	1.49 x 10^-54		4.53 10^-17	0
MCTS	4.54 10^-138	4.53 10^-17		0
VS-Chemprop	0	0	0	

Values shown are the calculated p-values (Bonferroni correction applied) between each generative model and the virtual screening approach for discovering hit compounds (antibacterial prediction score ≥ 0.5 and log solubility ≥ -4). Black cells are self-comparisons and cannot be calculated. Zero values represent p-values too small to be calculated.

Appendix Table S2. Bacterial counts 24-hpi for Vehicle and Treatment skin samples.

Vehicle (10% DMSO) Bacterial Burden 24-hpi (CFU/g)	Treatment (2% Synthecin) Bacterial Burden 24-hpi (CFU/g)
6214689266	46623794.21
7890743551	41522491.35
4656084656	35043804.76
5973715651	76131687.24
7822410148	69466882.07
