# Supplementary figures and images for: SyntheMol-RL: a flexible reinforcement learning framework for designing easily synthesizable antibiotics
Source: Mol Syst Biol. 2026 Apr 23;22(6):833–67. doi: 10.1038/s44320-026-00206-9 (PMC13230741; doi:10.1038/s44320-026-00206-9)

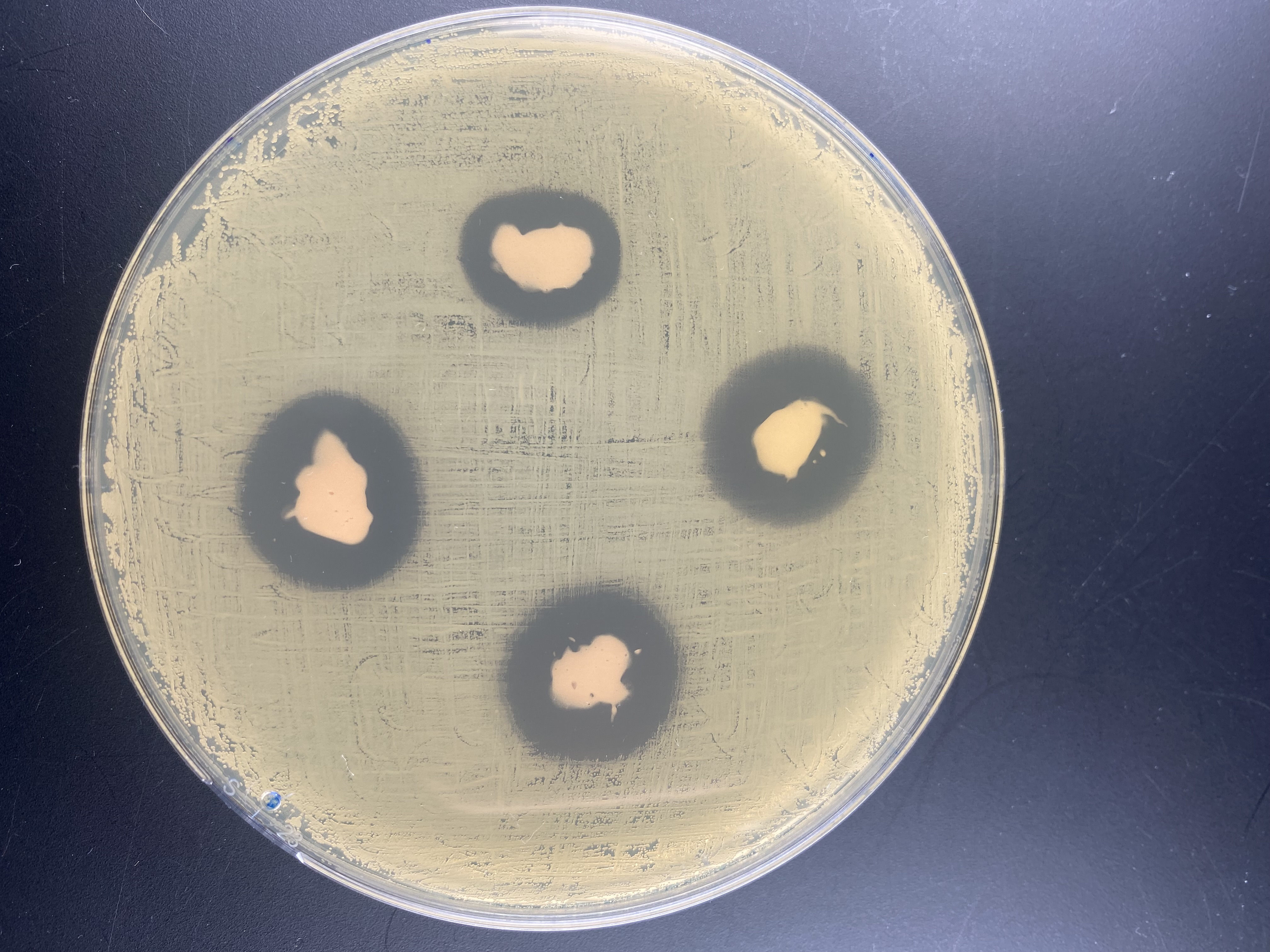

Supplement: Supplementary file 15 — Source data Fig. 5 [file 44320_2026_206_MOESM15_ESM.zip › Figure 5_source_data/5A/IMG_7013_raw.jpeg]

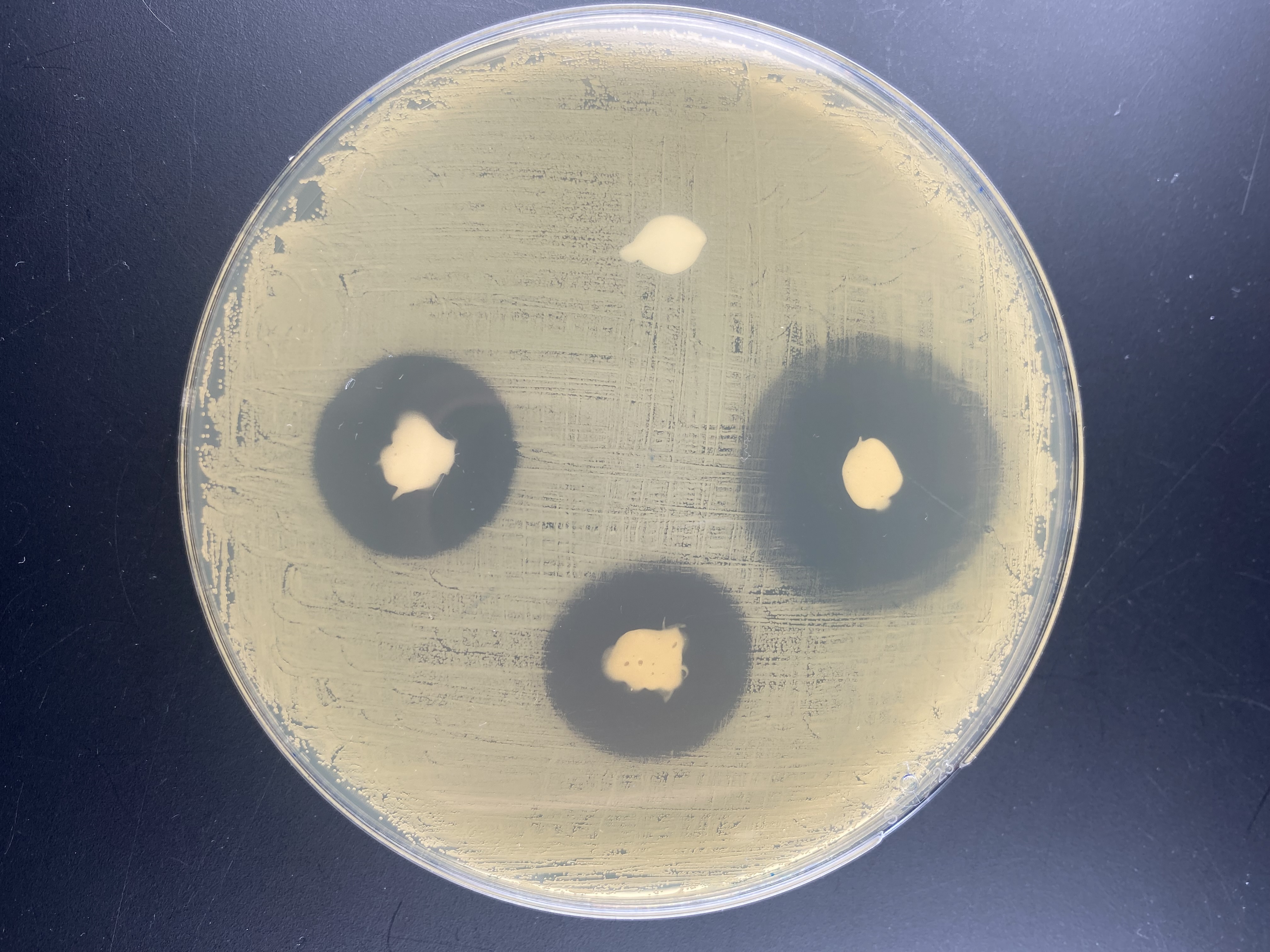

Supplement: Supplementary file 15 — Source data Fig. 5 [file 44320_2026_206_MOESM15_ESM.zip › Figure 5_source_data/5A/IMG_7019_raw.jpeg]
