# Supplementary material for: SyntheMol-RL: a flexible reinforcement learning framework for designing easily synthesizable antibiotics
Source: Mol Syst Biol. 2026 Apr 23;22(6):833–67. doi: 10.1038/s44320-026-00206-9 (PMC13230741; doi:10.1038/s44320-026-00206-9)
Supplement: Supplementary file 16 — Expanded View Figures [file 44320_2026_206_MOESM16_ESM.pdf]

Expanded View Figures

Figure EV1. Analysis of *S. aureus* property predictor model and chemical spaces.

(**A**) Normalized growth of *S. aureus* RN4220 in duplicate experiments used as the training data. (**B**) ROC and (**C**), precision–recall curves for each *S. aureus* activity property prediction model in an ensemble of ten, split by scaffold. Dark curves represent the average across all models. Area under the curve is indicated on the respective graphs. (**D**) Density plots showing the distribution of molecular weight and cLogP across (1) the *S. aureus* model training set, (2) a collection of known antibiotics from ChEMBL, and (3) the Enamine REAL and WuXi GalaXi chemical spaces explored by SyntheMol-RL. Not visualized are two molecules with molecular weights exceeding >3000 g/mol. (**E**) Same as (**D**), but focused on the region of the distribution containing the majority of molecules for visual clarity.

© The Author(s)

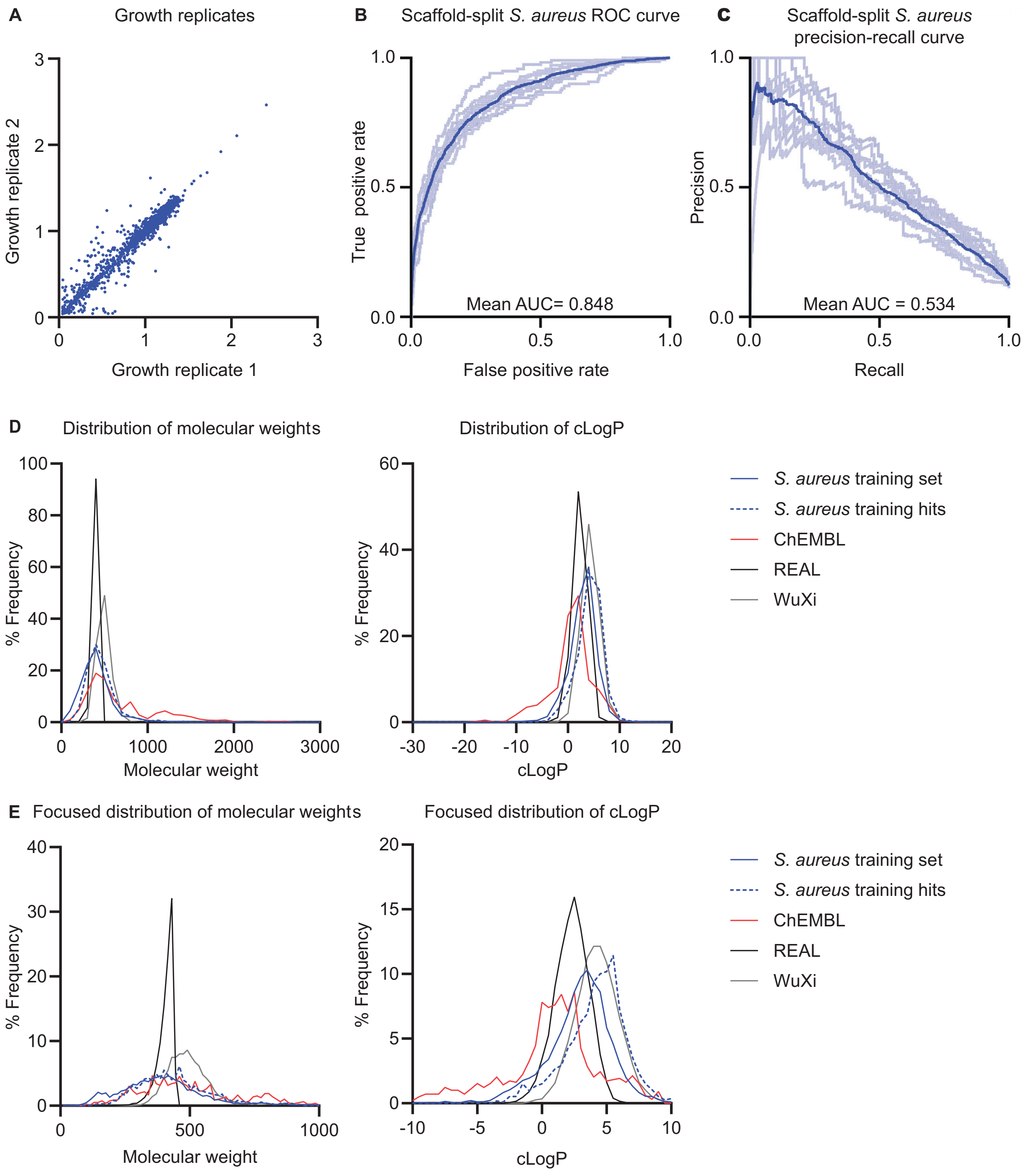
A
Growth replicates
Growth replicate 2
Growth replicate 1
0
1
2
3
B
Scaffold-split S. aureus ROC curve
True positive rate
False positive rate
Mean AUC= 0.848
0.0
0.5
1.0
C
Scaffold-split S. aureus precision-recall curve
Precision
Recall
Mean AUC = 0.534
D
Distribution of molecular weights
% Frequency
Molecular weight
0
20
40
60
80
100
1000
2000
3000
Distribution of cLogP
cLogP
-30
-20
-10
10
20
S. aureus training set
S. aureus training hits
ChEMBL
REAL
WuXi
E
Focused distribution of molecular weights
30
500
Focused distribution of cLogP
-5
5
15

 © The Author(s)

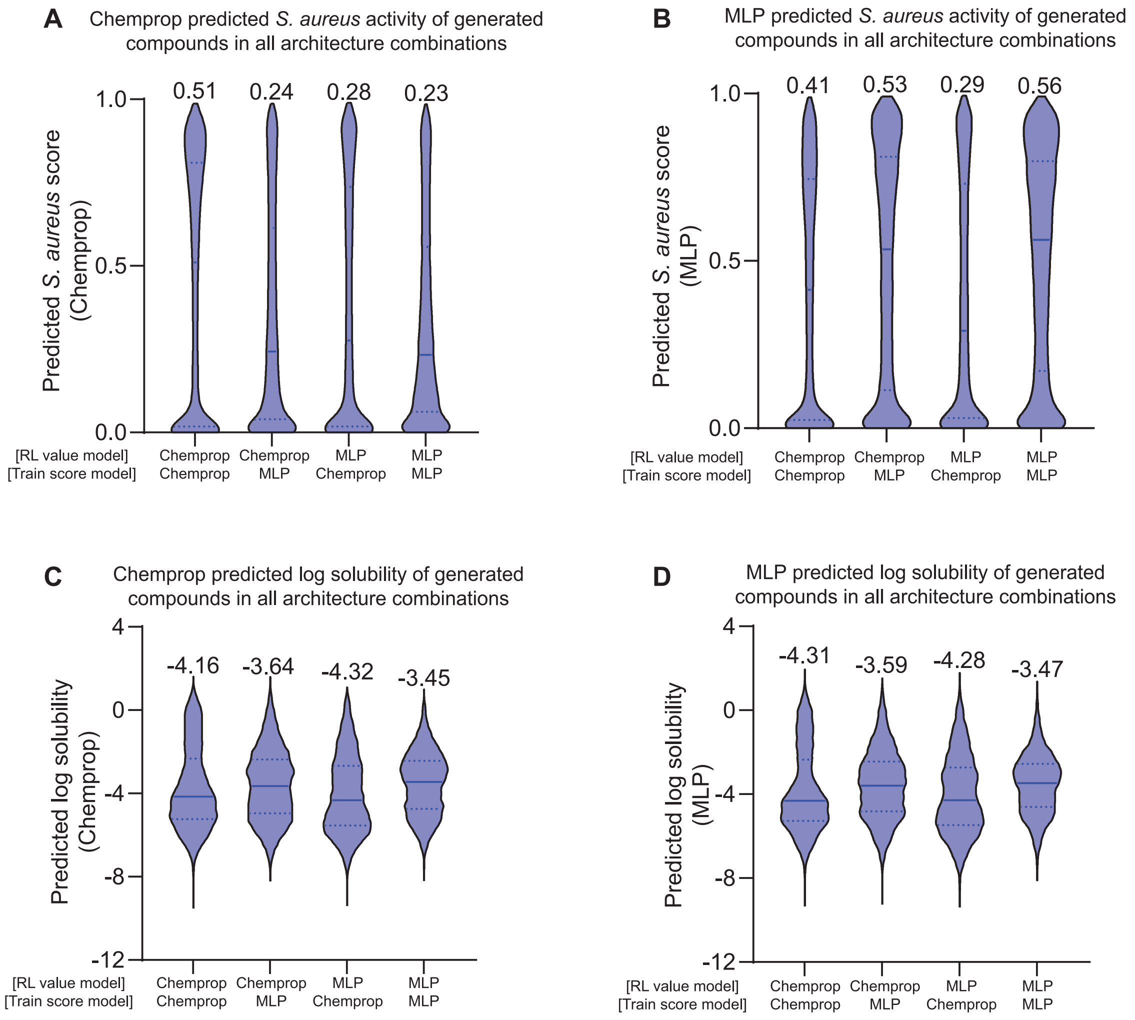

Figure EV2. Effects of architecture combinations on generated compound scores.

(**A**) Violin plots displaying the distribution of *S. aureus* scores evaluated by a Chemprop-RDKit model after generation across all combinations of deep learning architectures acting as the RL value model and the score model used for training it during generation. (Left to right, for all panels, $n = 10{,}983$; 10,534; 9228; 11,433.) (**B**) Violin plots displaying the distribution of *S. aureus* scores evaluated by an MLP-RDKit model after generation across all combinations of deep learning architectures acting as the RL value model and the score model used for training it during generation. (**C**) Violin plots displaying the distribution of log solubility scores evaluated by a Chemprop-RDKit model after generation across all combinations of deep learning architectures acting as the RL value model and the score model used for training it during generation. (**D**) Violin plots displaying the distribution of log solubility scores evaluated by an MLP-RDKit model after generation across all combinations of deep learning architectures acting as the RL value model and the score model used for training it during generation.

© The Author(s)

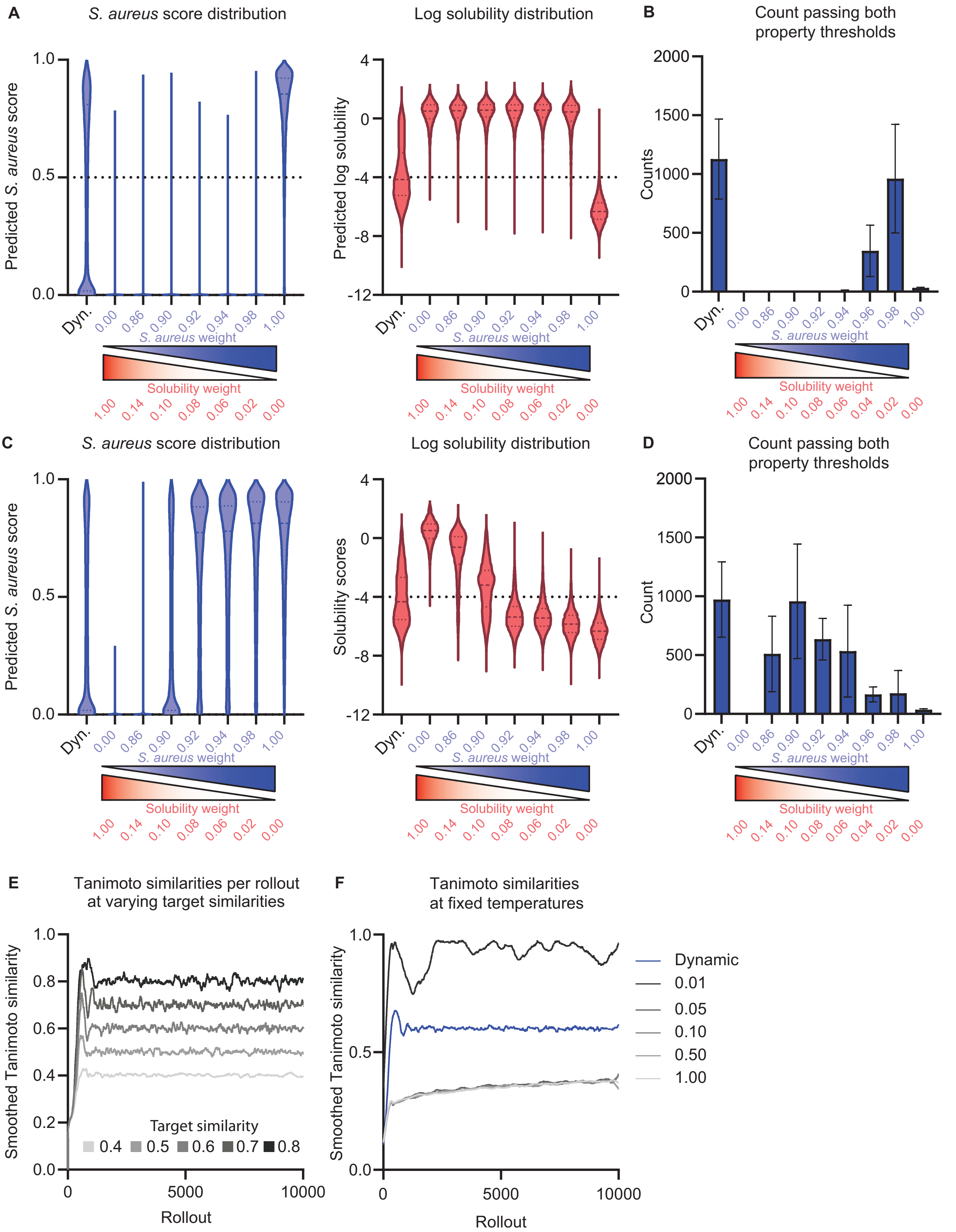
A
S. aureus score distribution
Predicted S. aureus score
Log solubility distribution
Predicted log solubility
B
Count passing both property thresholds
Counts
Dyn.
S. aureus weight
Solubility weight
C
S. aureus score distribution
Log solubility distribution
Solubility scores
D
Count passing both property thresholds
Count
E
Tanimoto similarities per rollout at varying target similarities
Smoothed Tanimoto similarity
Target similarity
0.4 0.5 0.6 0.7 0.8
Rollout
F
Tanimoto similarities at fixed temperatures
Dynamic
0.01
0.05
0.10
0.50
1.00

 © The Author(s)

Figure EV3. SyntheMol-RL ablation experiments.

(**A**) Distribution of predicted *S. aureus* activity and log solubility scores for the final dynamic weighting system (Dyn) and fixed weightings using RL-Chemprop. The sum of weights is equal to one. Left to right, for (**A**, **C**), $n = 10{,}537$; 10,282; 10,169; 10,484; 10,343; 10,613; 11,187. (**B**) Number of molecules generated that pass property "hit" thresholds for *S. aureus* activity and log solubility (≥0.5 and ≥ −4, respectively). Dynamic weighting achieves optimal or near-optimal performance without need for additional fine-tuning, shown by generating the most "hit" compounds. Error bars represent the range of values found across five differently seeded runs at each weighting. (**C**) Distribution of predicted *S. aureus* activity and log solubility scores for the final dynamic weighting system and fixed weightings using RL-MLP. The sum of weights is equal to one. (**D**) Number of molecules generated that pass property "hit" thresholds for *S. aureus* activity and log solubility (≥0.5 and ≥ −4, respectively). Error bars represent the range of values found across 5 differently seeded runs at each weighting. (**E**) Tanimoto similarities of each rollout compared to previous rollouts for RL-Chemprop set at desired target similarities in the range [0.4, 0.8]. The model can effectively generate compounds at a user-defined diversity. (**F**) Tanimoto similarities of each rollout compared to previous rollouts for RL-Chemprop at fixed temperatures and dynamic temperature for reference. Similarity is highly sensitive to changes in temperature, further necessitating the use of dynamic changes to output desired diversity.

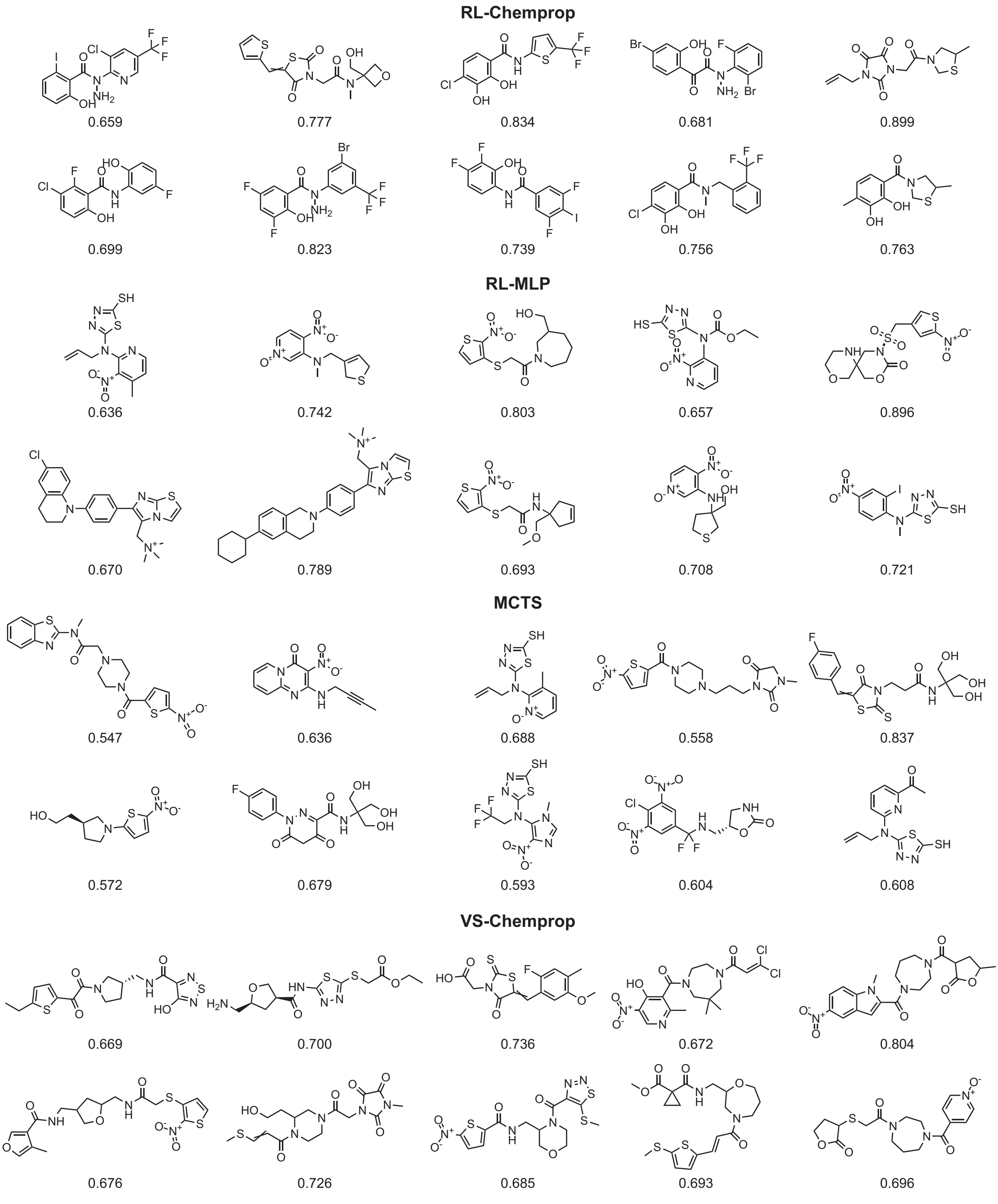

Figure EV4. Top compounds passing our novelty and diversity filters, randomly chosen from SyntheMol and virtual screening.

Ten random compounds from both SyntheMol-RL models, SyntheMol-MCTS, and VS-Chemprop were selected at the final in silico filtering stage "Top 150 novel diverse hits". Each compound is labeled with its respective predicted *S. aureus* score.

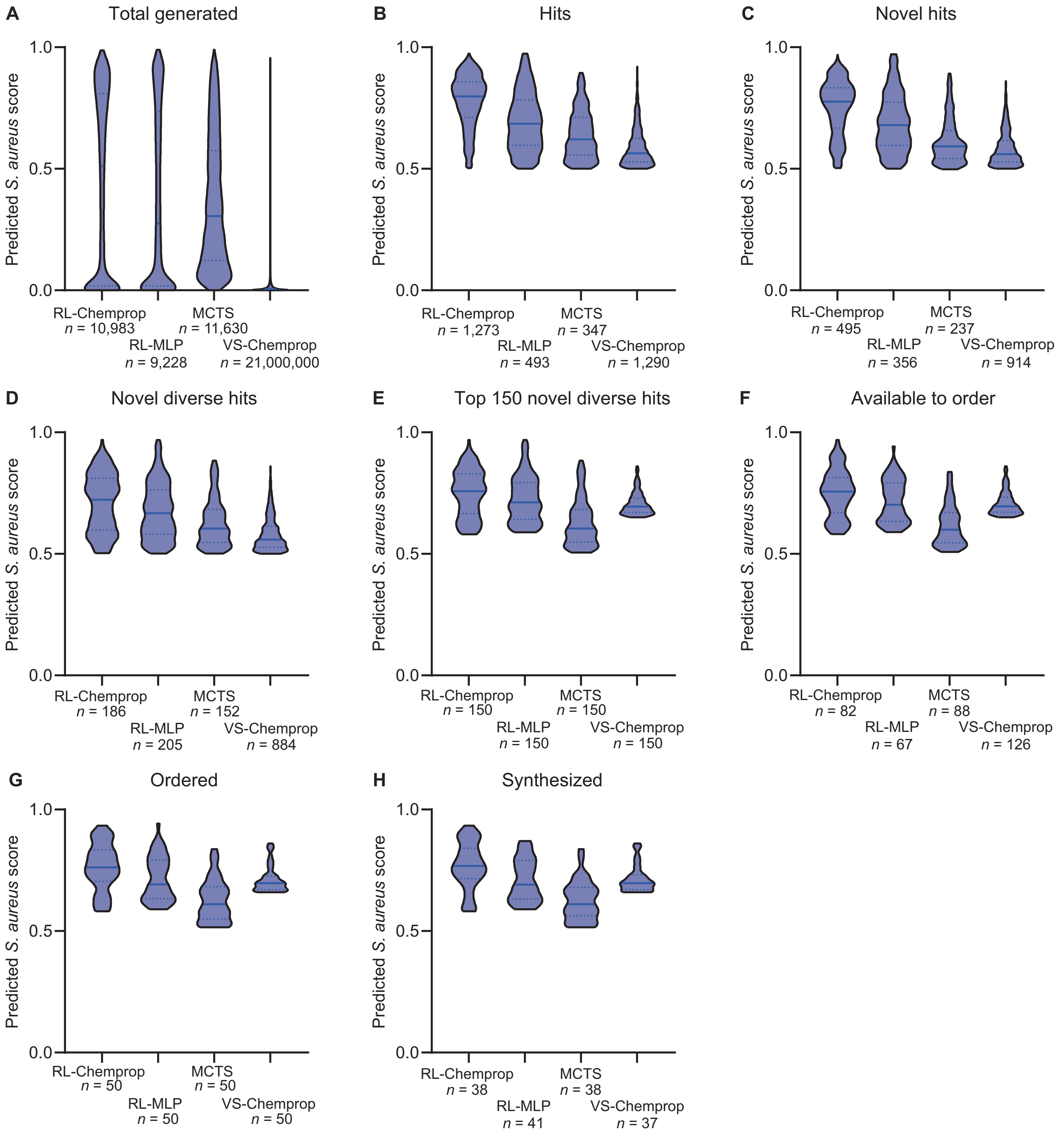

Figure EV5. Distribution of predicted *S. aureus* activity from SyntheMol and virtual screening at each post-hoc filtering stage.

(**A**) All molecules generated or screened by each method. (**B**) Molecules from (**A**) with predicted *S. aureus* score ≥0.5 and predicted log solubility ≥ −4. This filter has the greatest impact on the predicted *S. aureus* score distribution, with the remaining filters having minimal impact. (**C**) Molecules from (**B**) that have a maximum Tversky similarity ≤0.6 compared to all known antibiotics in the training set and the ChEMBL antibiotics. (**D**) Molecules from (**C**) that have a maximum Tanimoto similarity to other selected molecules ≤0.6. (**E**) Molecules from (**D**) with the top 150 predicted *S. aureus* scores. (**F**) Molecules from (**E**) that are available to order from Enamine or WuXi. (**G**) Molecules from (**F**) with the lowest 50 predicted clinical toxicity values, which were ordered from Enamine or WuXi. (**H**) Molecules from (**G**) that were successfully synthesized by Enamine or WuXi and were experimentally tested.

© The Author(s)

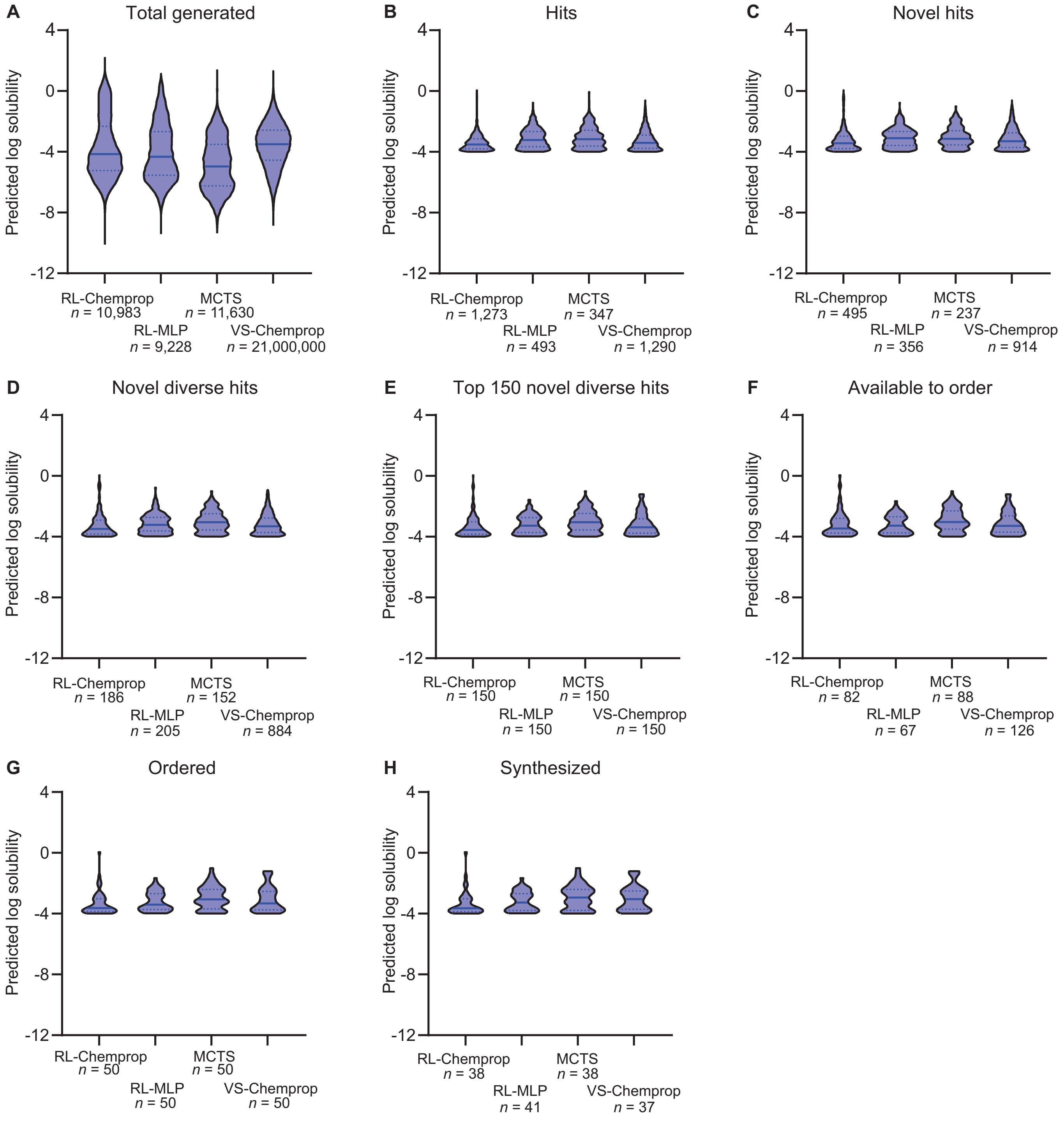

Figure EV6. Distribution of predicted log solubility from SyntheMol and virtual screening at each post-hoc filtering stage.

(**A**) All molecules generated or screened by each method. (**B**) Molecules from (**A**) with predicted *S. aureus* score ≥0.5 and predicted log solubility ≥ −4. This filter has the greatest impact on the predicted log solubility distribution, with the remaining filters having minimal impact. (**C**) Molecules from (**B**) that have a maximum Tversky similarity ≤0.6 compared to all known antibiotics in the training set and the ChEMBL antibiotics. (**D**) Molecules from (**C**) that have a maximum Tanimoto similarity to other selected molecules ≤0.6. (**E**) Molecules from (**D**) with the top 150 predicted *S. aureus* scores. (**F**) Molecules from (**E**) that are available to order from Enamine or WuXi. (**G**) Molecules from (**F**) with the lowest 50 predicted clinical toxicity values, which were ordered from Enamine or WuXi. (**H**) Molecules from (**G**) that were successfully synthesized by Enamine or WuXi and were experimentally tested.

 © The Author(s)

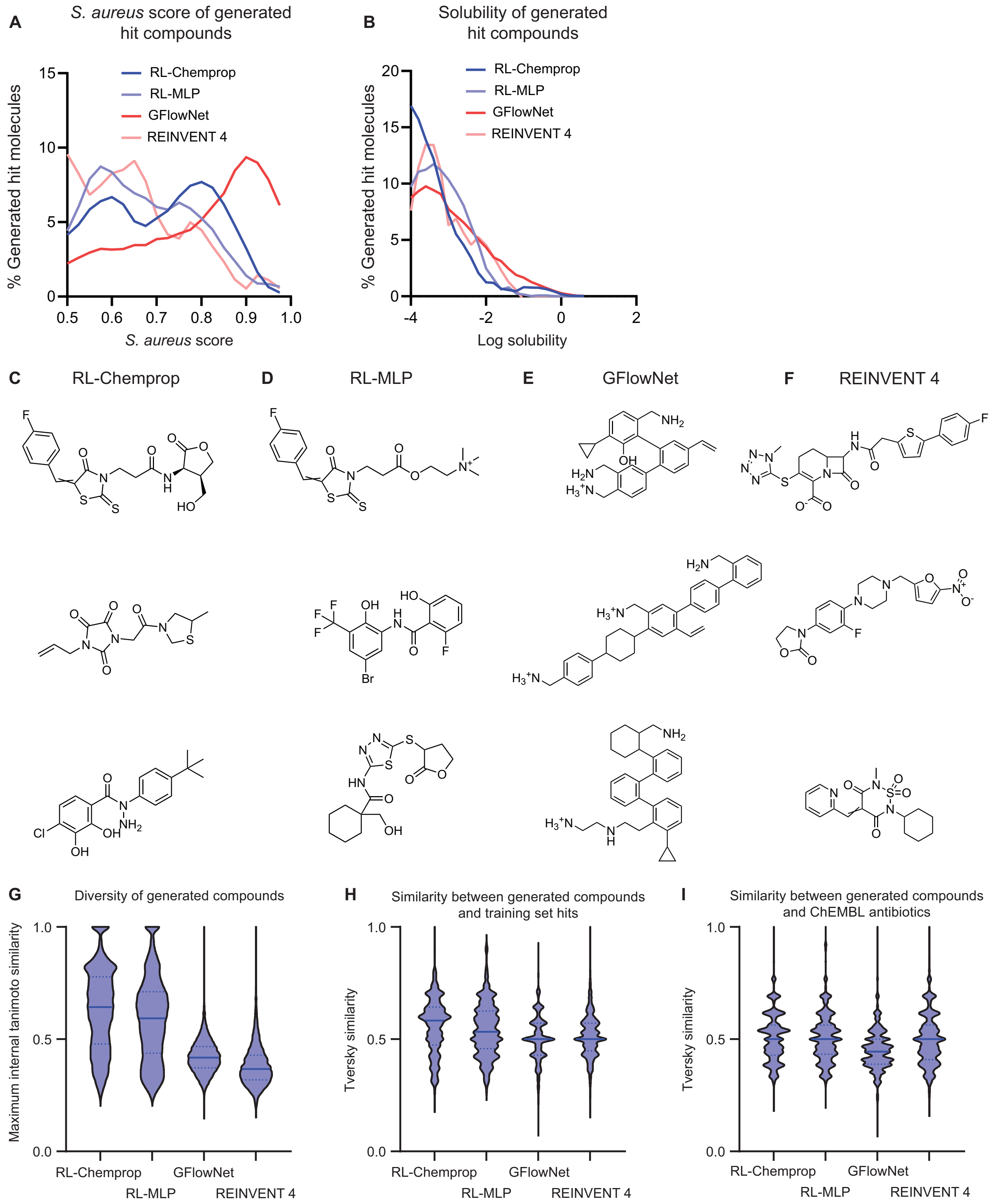
A
S. aureus score of generated hit compounds
RL-Chemprop
RL-MLP
GFlowNet
REINVENT 4
% Generated hit molecules
S. aureus score
B
Solubility of generated hit compounds
% Generated hit molecules
Log solubility
C
RL-Chemprop
D
RL-MLP
E
GFlowNet
F
REINVENT 4
G
Diversity of generated compounds
Maximum internal tanimoto similarity
H
Similarity between generated compounds and training set hits
Tversky similarity
I
Similarity between generated compounds and ChEMBL antibiotics
Tversky similarity

Figure EV7. Comparisons of compounds generated by SyntheMol-RL, GFlowNet, and REINVENT 4.

SyntheMol-RL, GFlowNet, and REINVENT 4 generated "hit" compounds that passed filters for predicted antibacterial activity (≥0.5), log solubility (≥ −4), novelty, and diversity ("Methods"). Additionally, GFlowNet and REINVENT 4 molecules were filtered for synthesizability (synthetic accessibility score SAScore ≤4) and molecular weight (weight ≤600) to ensure a fair comparison to SyntheMol-RL compounds, which are intrinsically synthetically accessible and small. RL-Chemprop generated 186 hits (total = 10,983), RL-MLP generated 205 hits (total = 9228), GFlowNet generated 1152 hits (total = 10,304), and REINVENT 4 generated 36 hits (total = 9840). Total generated compounds are further analyzed in (**G**–**I**). These hits were compared based on two properties of interest: (**A**) *S. aureus* score, and (**B**) log solubility score. Representative molecules are visualized for (**C**) RL-Chemprop, (**D**) RL-MLP, (**E**) GFlowNet, and (**F**) REINVENT 4. See Appendix Extended Discussion. (**G**) Violin plots showing the distribution of maximum Tanimoto similarities to other generated molecules in the same set for measuring structural diversity of generated compounds. (**H**) Violin plots showing the distribution of Tversky similarity calculated between generated compounds and hits from the *S. aureus* training dataset. (**I**) Violin plots showing the distribution of Tversky similarity calculated between generated compounds and the ChEMBL antibiotics.

 © The Author(s)

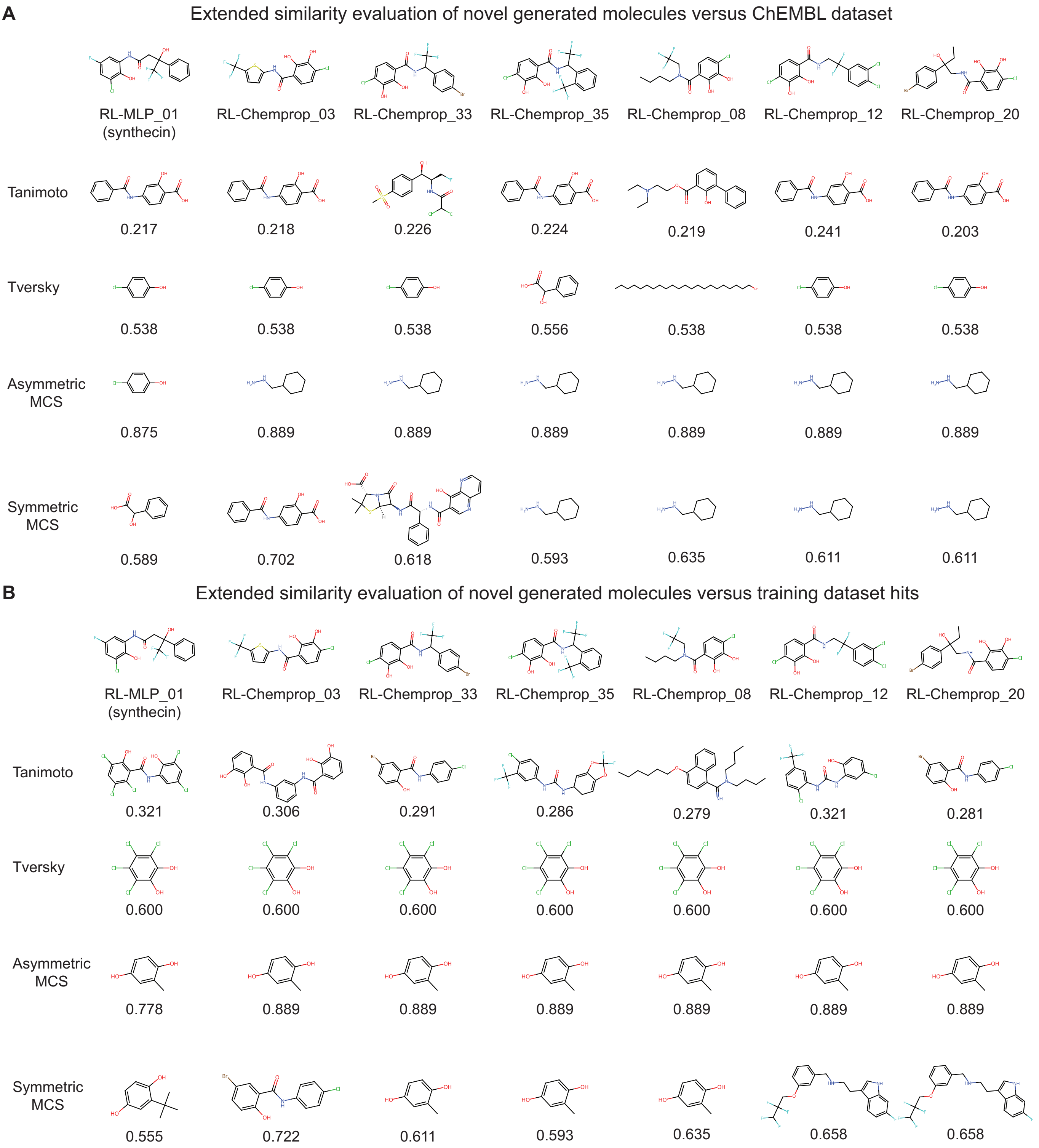

Figure EV8. Similarity of generated compounds compared to ChEMBL antibiotics.

(**A**) Molecules in the top row are highly potent, generated compounds that have passed a manual literature search for novelty using resources such as SciFinder. Each row below represents the most similar compound among the known ChEMBL antibiotics, along with the quantified similarity value. In total, four metrics of similarity were used: Tanimoto on Morgan fingerprints, Tversky on Morgan fingerprints, asymmetric Maximum Common Substructure (MCS) ratio, and symmetric Maximum Common Substructure (MCS) ratio. (**B**) Molecules in the top row are highly potent, generated compounds that have passed a manual literature search for novelty using resources such as SciFinder. Each row below represents the most similar hit compound in the training dataset along with the quantified similarity value. In total, four metrics of similarity were used: Tanimoto on Morgan fingerprints, Tversky on Morgan fingerprints, asymmetric Maximum Common Substructure (MCS) ratio, and symmetric Maximum Common Substructure (MCS) ratio.

© The Author(s)

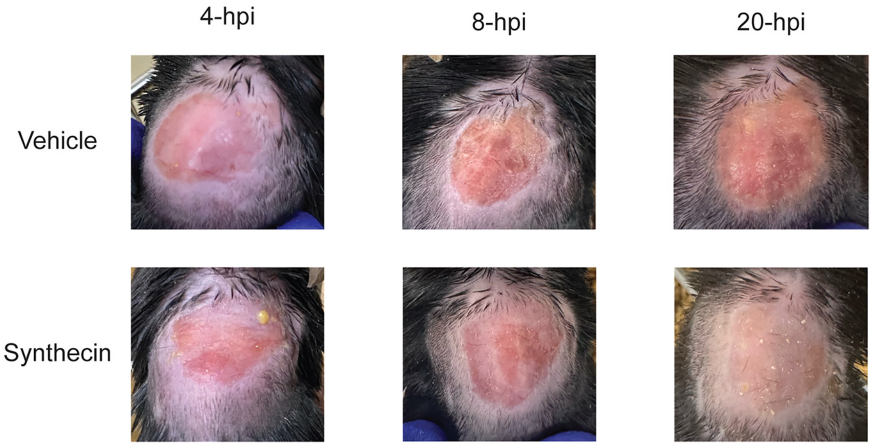

Figure EV9. Dorsal surface of mice with an *S. aureus* wound infection model.

Representative images of the dorsal surface of mice after 4 h, 8 h, and 20 h of treatment with vehicle or synthecin. Note the marked inflammation on the vehicle-treated animals.

 © The Author(s)

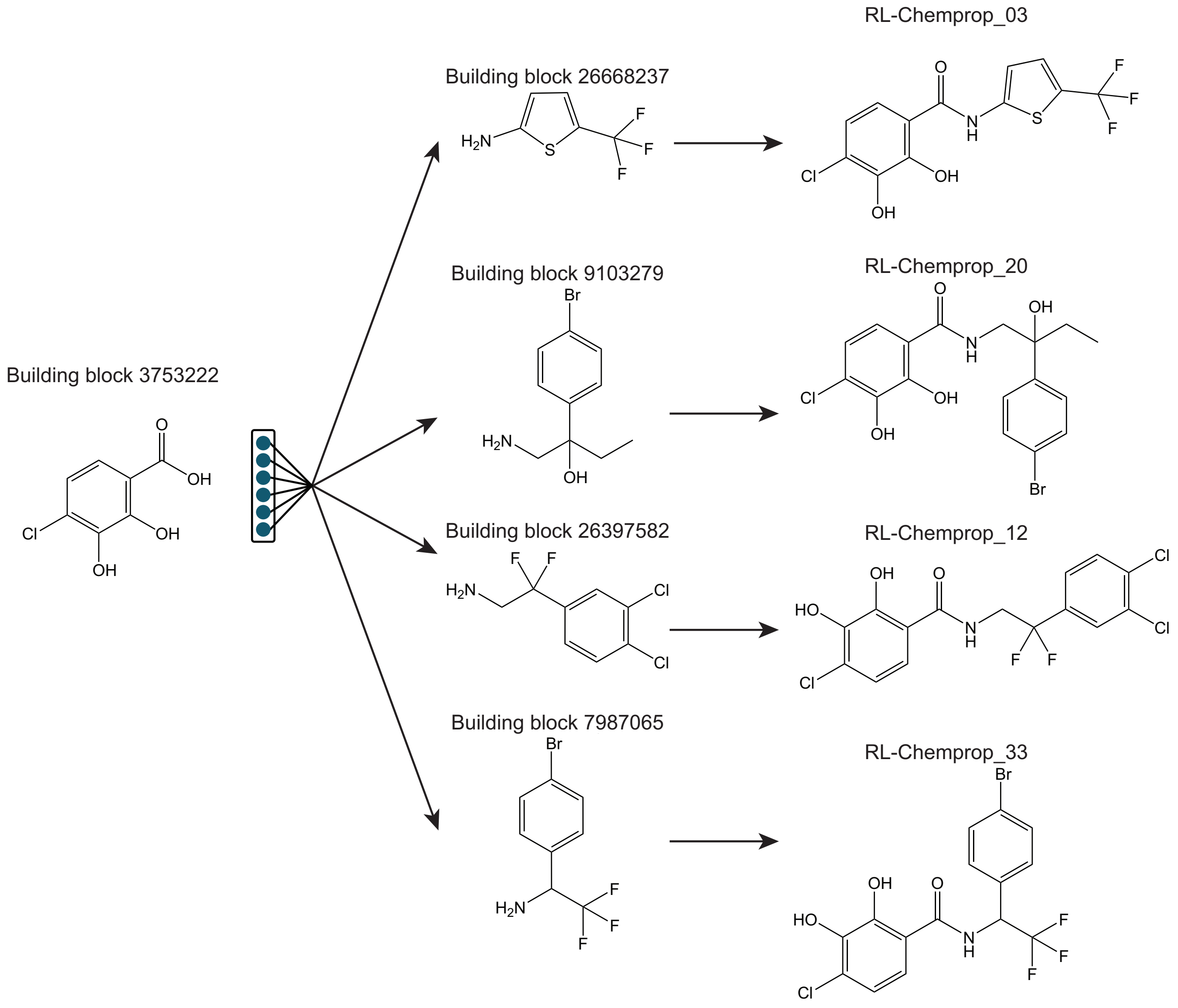

Figure EV10. Example of molecular generations centered around particular building blocks.

Depicted is building block 3753222, which SyntheMol-RL used multiple times when constructing hit compounds. Notably, all four compounds shown were validated to have an MIC ≤ 8 µg/ml in *S. aureus* RN4220. SyntheMol-RL seemingly employs a strategy of finding a particularly promising building block, then to satisfy the diversity objective it expands on that building block with a diverse set of “secondary” building blocks.

© The Author(s)
